# Genetic characterization and phylogenetic relationships of *Phyllodistomum* parasites in Indian subcontinent: insights from freshwater fish and shrimp hosts

**DOI:** 10.1007/s00436-023-07930-3

**Published:** 2023-08-23

**Authors:** Kirti Choudhary, Shailendra Ray, Nirupama Agrawal, Shokoofeh Shamsi

**Affiliations:** 1 Department of Zoology, University of Lucknow, Lucknow, U.P. 226007 India; 2 School of Agricultural, Environmental and Veterinary Sciences, Gulbali Institute, Charles Sturt University, Wagga Wagga, Australia

**Keywords:** *Phyllodistomum*, Internal transcribed spacer, Intra-specific variation, Metacercaria

## Abstract

*Phyllodistomum* is the large digenean group of fish parasites, with 25 species described so far in the Indian subcontinent. Here, we redescribed two adult species of *Phyllodistomum* (*P. srivastava* Rai 1964 and *P. parorchium* Jaiswal 1957) collected from freshwater fish *Heteropneustes fossilis* Bloch, 1974 and *Glossogobius giuris* Ham, 1822, respectively, and an unknown *Phyllodistomum* metacercaria from shrimp (*Macrobrachium dayanum* Henderson, 1893). These parasites were genetically characterized using 28S and first and second internal transcribed spacers (ITS1 and ITS2) regions of the nuclear ribosomal DNA and CoxI region of the mitochondrial (mt) DNA to establish the link between metacercaria and adult. Morphologically, both the unknown metacercaria in shrimp and adult *Phyllodistomum srivastava* in fish, resembled in terms of crenulated margin of hind body, arrangement of diagonal testes, bipartite seminal vesicle, and compact paired vitelline masses. The two adult parasite species, *P. srivastava* from *P. parorchium*, were different in terms of shape and size of the body, ratio of suckers, the absence of crenulated margin of hind body, a single chambered seminal vesicle, and deeply lobed paired vitelline masses, in the former species. Comparison of the 28S, ITS, and mtCoxI sequence data suggested *P. srivastava* and *Phyllodistomum* metacercaria belong to the same species, and supported the distinction between *P. srivastava* and *P. parorchium*. Exploring the potential impact of *Phyllodistomum *infection on host behaviour and health would be prospective areas for future research.

**Supplementary Information:**

The online version contains supplementary material available at 10.1007/s00436-023-07930-3.

## Introduction

The genus *Phyllodistomum* Braun 1899 is a large group of digenean fish parasites, comprising 120 species, under the family Gorgoderidae, Looss 1901. *Phyllodistomum* spp. are known to parasitize both marine and freshwater fishes, with occasional reports of infestations in amphibians (Campbell 2008). They have global distribution. The genus *Phyllodistomum* is characterized by broad, tapering fore body and foliate hind body with more or less crenulated body margin (Campbell 2008). Of the 120 species belonging to the genus *Phyllodistomum*, 25 nominal species, distinguished by conventional morphological characters, are so far reported from freshwater fish in the Indian subcontinent (Table 1) (Jaiswal 1957; Rai 1964; Sarwat 2011; Naz and Siddique 2012; Sen 2014). In previous studies, Pandey (1970) and Rai (1964) collected phyllodistome metacercariae encysted in the hepatopancreas of crustaceans, *Macrobrachium dayanum* Henderson (1893) and obtained their adults, by performing feeding experiments in fish, *Heteropneustes fossilis.* They identified these phyllodistomes as *P. srivastava* and *P. lucknowensis.*

**Table 1:** Previous reports of *Phyllodistomum* in India. Scientific names of the host are based on the currently accepted scientific names (Froese and Pauly 2019)

Parasite species	Host species	Infected organ	Locality in India	Reference
*P. betwaensis*	*Channa punctatus* (Bloch)	Intestine	Betwa River, Bundelkhand region, Jhansi	Sen (2014)
*P. bimaculatus*	*Ompok bimaculatus* (Bloch)	Stomach	Varanasi	Ahmad et al. (1999)
*P. cameroni*	*Rita rita* (Hamilton)	Intestine	Lucknow	Agrawal (1966)
*P. cephaloglandulatum*	*Mastacembelus armatus* (Lacepède)	Urinary Bladder	Faizabad	Pande and Dwivedi (1983)
*P. chauhani*	*Sperata aor* (Hamilton), *Sperata seenghala* (Sykes)	Urinary bladder	Allahabad	Motwani and Srivastava (1961)
*P. chitala*	*Chitala chitala* (Hamilton)	Stomach	Gwalior (M.P.), India	Bhadauria and Dandotia (1988)
*P. folium*	*Glyptosternon* sp. McClelland (*Glyptosternum*)	Urinary bladder	Muzaffarnagar	Kakaji (1969)
*P. guptai*	*Clarias batrachus* (Linnaeus), *Heteropneustes fossilis* (Bloch), *Channa gachua* (Hamilton)	Body cavity	Loktak Lake, Bishnupur district, Manipur	Shomorendra and Jha (2006)
*P. indianum*	*Heteropneustes fossilis* (Bloch)	Cloaca	Hyderabad	Jaiswal (1957)
*P. lewisi*	*Strongylura strongylura* (van Hasselt)	Urinary bladder	Allahabad River, Ganges & Yamuna	Srivastava (1938)
*P. longicephalus*	*Setipinna phasa* (Hamilton)	Urinary bladder	Danapur (Patna)	Singh and Sinha (1975)
*P. loossi*	*Schizothorax esocinus* (Heckel)	Urinary bladder	Kashmir	Kaw (1950)
*P. lucknowensis*	*Heteropneustes fossilis* (Bloch)	Intestine	Lucknow	Pandey (1970)
*P. macrobius*	*Mystus tengara* (Hamilton)	Body cavity	Lucknow	Yamaguti (1958)
*P. parichhaii*	*Xenentodon cancila* (Hamilton)	Intestine	Bundelkhand region, Jhansi	Naz and Siddiqui, 2012
*P. parorchium*	*Glossogobius giuris* (Hamilton)	Body cavity	Hyderabad	Jaiswal (1957)
*P. singhiai*	*Mastacembelus armatus* (Lacepède)	Intestine	Lucknow	Gupta (1951)
*P. spatulaeformae*	*Monopterus cuchia* (Hamilton) (*Amphipnous cuchia*)	Urinary bladder	Muzaffarnagar	Kakaji (1969)
*P. srivastava*	*Heteropneustes fossilis* (Bloch)	Urinary bladder	Jabalpur	Rai (1964)
*P. triangulata*	*Mastacembelus armatus* (Lacepède)	Intestine	Paithan, Aurangabad, (M.S.)	Sarwat (2011)
*P. tripathi*	*Bagarius bagarius* (Hamilton)	Urinary bladder	Allahabad	Motwani and Srivastava (1961)
*P. vachius*	*Eutropiichthys vacha* (Hamilton)	Urinary bladder	Lucknow	Dayal (1949)
*P. vittatus*	*Macrones vittatus* (Bloch)	Intestine	Guwahati (Assam)	Gupta (1955)
*P. pahujii*	*Xenentodon cancila* (Hamilton)	Intestine	Bundelkhand region, Jhansi	Naz and Siddiqui (2012)
*Phyllodistomum* sp.	*Labeo fimbriatus* (Bloch)	Body cavity	Hyderabad	Jaiswal (1957)

Digeneans commonly undergo a series of developmental stages involving different hosts, making their life cycles intricate and challenging to study solely through traditional methods (Aghlmandi et al. 2018). Sequence data have proven to be invaluable in elucidating the life cycles of digeneans, which typically exhibit an indirect life cycle. The use of genetic information, such as DNA sequencing of specific regions like 28S rRNA, ITS1, and CoxI, has provided crucial insights into the complex life cycles of these parasitic flatworms (Huston et al. 2018; Rochat et al. 2020; Shamsi et al. 2021a; Shamsi et al. 2021b; Shamsi et al. 2023, Barton et al. 2022; Choudhary et al. 2022).

Cribb (1987) highlighted that the proper identification of *Phyllodistomum* spp. is challenging due to significant intra-specific morphological variations and inadequate descriptions of several species. However, recent advances in molecular biology, particularly PCR and sequencing-based molecular techniques, have proven effective in identifying and distinguishing digenean parasites (Blair et al. 1996; Hust et al. 2004; Goswami et al. 2009). These molecular techniques have been particularly useful in elucidating the taxonomic status of *Phyllodistomum* species from various parts of the world. For instance, molecular studies have successfully identified and distinguished species such as *P. centropomi, P. lacustri*, *P. inecoli, P. cribbi*, *P. wallacei*, and *P. spinopapillatum* from Mexico; *P. kanae*, *P. parasiluri*, and *P. elongatum* from Japan; *P. folium* from Spain; *P*. *pseudofolium* and *P. angulatum* from Lithuania and Russia; *P. magnificum* and *P. symmetrochis* from Australia; and *P. staffordi* and *P. brevicaceum* from Canada (Mendoza-Garfias and de León 2005; Peribáñez et al. 2011; Cutmore et al. 2013; Razo-Mendivil et al. 2013; de León et al. 2015a; de León et al. 2015b; Nakao 2015; Urabe et al. 2015; Stunžėnas et al. 2017). However, the molecular study of *Phyllodistomum* species from India remains entirely unexplored. The ribosomal DNA (rDNA) is helpful in resolving the phylogeny because it is universal and composed of highly conserved as well as variable domains (Tkach et al. 2000; Mwita and Nkwengulila 2010). The internal transcribed spacer regions (ITS1 and ITS2) also include a high degree of inter- and intra-specific genetic variations (Nolan and Cribb 2005; Littlewood 2008; Choudhary et al. 2015). Additionally, the mitochondrial cytochrome oxidase 1 (Cox1) has also been used for distinguishing digenean species and inferring phylogenies (Georgieva et al. 2013). Therefore, by coupling molecular and morphological approaches, uncertainties of existing species could be resolved. Also, the preliminary identification and molecular data allow establishing a link between metacercaria found in shrimp and adult in freshwater fish, not yet elucidated in India.

We hereby present first molecular data to (1) distinguish and identify two *Phyllodistomum* species and (2) correlate one with its metacercaria, in combination with sequences, and (3) to determine the systematic position.

## Materials and methods

### Parasite collection and identification

**Figure 1:**
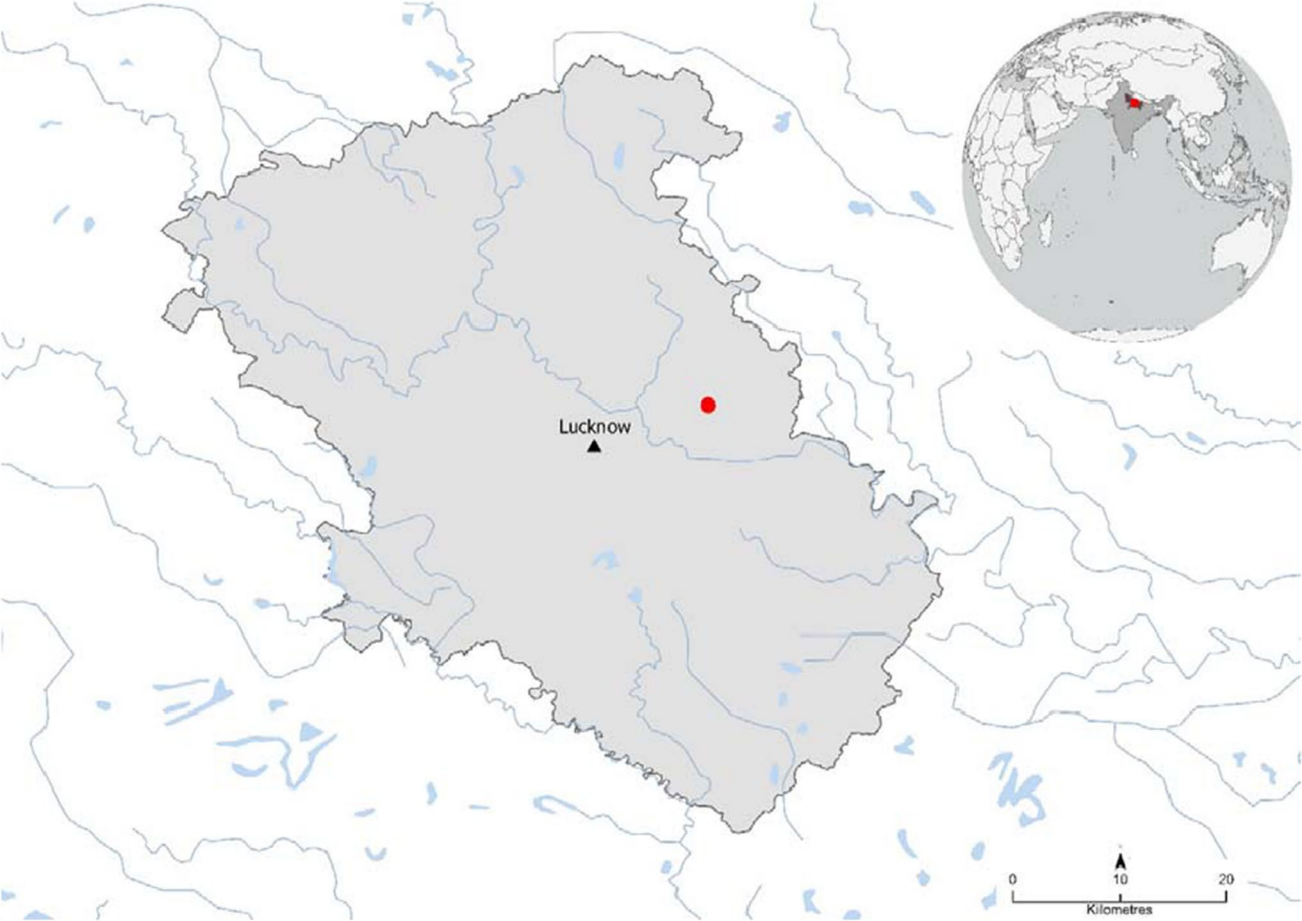
Study area in the present study

During winter months (November to January 2016), 65 live specimens of freshwater fish and shrimp were captured from the Gomti River, Lucknow, India (Fig. 1). To detect adult *Phyllodistomum* infection, the fish were euthanized through spinal severance and then examined under a stereomicroscope. In addition, numerous specimens of naturally infected *Phyllodistome* metacercariae from the hepatopancreatic tissue of the freshwater shrimp were collected. This work was conducted under animal ethic approval 20/I/2023/IAEC/LU.

### Morphological study

The recovered worms were examined under a light microscope and identified using references from the literature (Yamaguti 1934; Pandey and Agrawal 2013; Pandey and Agrawal 2018). Once identified, they were fixed in 70% ethanol for whole mount preparations, and a small piece was preserved in absolute ethanol for DNA extraction. Permanent mounts were prepared by staining the fixed whole mounts in aceto-alum carmine. Subsequently, the specimens were dehydrated through a graded ethanol series (50%, 70%, 90%, and absolute ethanol), cleared in clove oil, and then mounted on glass slides using DPX mounting medium. Figures were illustrated by drawing tube, attached to a phase-contrast light microscope (Olympus BX-51). All morphometric measurements (in millimeters) were taken with the aid of ocular micrometer. Voucher specimens were deposited at Helminthological Collection of the Zoological Survey of India, Kolkata.

### Molecular study

DNA was extracted using Qiagen’s DNeasy Blood and Tissue Kit according to the manufacturer protocols. Three nuclear ribosomal loci (28S, ITS1, ITS2) and one mitochondrial cytochrome oxidase subunit I (CoxI) were amplified using the following primer sets: 28S (Forward): 5′-ACCCGCTGAATTTAAGCAT-3′ and (Reverse): 5′-CTCTTCAGAG TACTTTTCAA-3′; ITS1 (Forward): 5′-GTCGTAACAAGGTTTCCGTA-3′ and 5′-TCTAGATGCGTTCGA(G/A)TGTCGATG-3′; ITS2 3S (Forward): 5′-GGTACCGTGGATCACTCGGCTCGTG-3′ and A28 (Reverse): 5′-GGGATCCTGGTTAGTTTCTTTTCCTCCGC-3′; and COI JB3 (Forward): 5′-TTTTTTGGGCATCCTGAGGTTTAT-3′ and JB4.5 (Reverse): 5′-TAAAGAACATAATGAAAATG-3′ primer (Bowles et al. 1993; Mollaret et al. 2000; Prasad et al. 2008). Each PCR amplification reaction is performed in a final volume of 12.5 μl, containing 10X buffer (100 mM Tris, pH 9.0), 50 mM KCl and 15mM MgCl2, 2.5 U Taq polymerase enzyme, 10 mM of each deoxynucleotide triphosphates (dNTPs), and 3μl DNA. The PCR conditions are as follows: initial denaturation at 94 °C for 5 min, annealing for 28S at 54 °C (1 min), ITS-1 at 54 °C (1.10 min), ITS-2 at 57 °C (1.10 min), mt CoxI at 56 °C (1.10 min), and final extension at 72 °C for 10 min. PCR products are checked on 2% agarose gel in TAE buffer, stained with ethidium bromide (EtBr) and visualized under UV light. The acquired PCR products were purified and subjected to Sanger sequencing in the forward direction, using an ABI3730xl DNA Analyzer (Applied Biosystems, Foster City, CA).

Similarity search analysis of nucleotide sequence was performed by Basic Local Alignment Search Tool (BLAST, https://blast.ncbi.nlm.nih.gov/Blast) and Clustal W (http://www.ebi.ac.uk/clustalw/) used for multiple sequence alignment. BioEdit software version 7.0.9.0 (Hall 1999) was used for sequence identities.

Phylogenetic trees were constructed (Table 2) using MEGA 11 (Tamura et al. 2013). Neighbor-joining (NJ) and maximum likelihood (ML) analysis were performed for each data set (28S, ITS1, ITS2, and mtCoxI). The best nucleotide substitution modal was estimated “Kimura 2-parameter model” for NJ tree and “General time reversible model” for ML trees with a gamma distribution of rates and proportion of invariant sites (GTR+G+I). The reliability of internal branches in all trees was evaluated by using the bootstrap method, 1000 replicates.

**Table 2:** Details of the GenBank accession numbers used to make phylogenetic trees in the present study. Asterisks denote sequences obtained in the present study.

Parasite species	Host	Locality	DNA region			
			28S	ITS 1	ITS2	CoxI
*P. angulatum*	*Sander lucioperca*	Russia	KX957734	KJ740511, KJ740512	KY307872	-
*P. brevicaecum*	*Umbra limi*	Canada	HQ325009, HQ325008, KC760204	KC760194, KC760195	-	KC760183
*P. centropomi*	*Centropomus parallelus*	Mexico	KM659384	-	-	KT376733
*P. cribbi*	-	Mexico	KT376718	-	-	KT376727, KT376731
*P. folium*	*Pisidium amnicum, Sphaerium corneum, Gasterosteus aculeatus*	Estonia, Russia, Lithuania	KJ729541, KJ729534	AY277704, AY277705	AY277705	-
*P. hoggettae*	*Plectropomus leopardus*	Australia	KF013191	-	KF013148	-
*P. hyporhamphi*	*Hyporhamphus australis*	Australia	KF013190	-	KF013150	-
*P. inecoli*	*Profundulus* sp., *Heterandria bimaculata, Poecilia sphenops*	Mexico	KM659389, KC760199, KM659383	KC760189, KC760188	-	KC760176, KC760169
*P. kanae*	*Hynobius retardatus*	Japan	AB979868	-	-	AB979869
*P. kupermani*	*Perca fluviatilis*	Russia	MT875008, MT875009	MT875012, MT875013	MT875012, MT875013	-
*P. lacustri*	*Ameiurus melas, Noturus flavus, Ictalurus pricei, Ictalurus dugesii*	USA, Canada, Mexico	EF032692, HQ325010	KC760198	-	HQ325044, HQ325040, HQ325045
*P. macrocotyle*	*Dreissena polymorpha, Leuciscus idus, Scardinius erythrophthalmus, Dreissena polymorpha*	Belarus, Russia, Lithuania	AY281127, MT872663, MT872664, AY288828	MT875011, AF533015, AY288831	MT875010, MT875011, AF533015, AY288831	-
*P. magnificum*	*Tandanus tandanus, *	Australia	KF013189, KF013186	KF013153	KF013156, KF013153	-
*P. pacificum*	*Pantolabus radiatus*	Australia	-	-	MG845601	-
*P. parasiluri*	*Silurus asotus*	Japan	LC002522	-	-	LC002524
*P. parorchium*	*Glossogobius giuris*	India (Present study)	MH047371*	MH047373*	OR269262*	OR420919
*P. pseudofolium*	*Gymnocephalus cernuus*	Russia	KX957732	-	KY307879	-
*P. spinopapillatum*	*Profundulus balsanus*, *Profundulus oaxacae*	Mexico	KM659382, KM659381	-	-	MW804304, MW804307, KT376732
*P. srivastava*	*Heteropneustes fossilis*	India (Present study)	MH047370*	MH047372*	OR240244*	OR420917
*P. staffordi*	*Ameiurus melas*	Canada	HQ325027	KC760197, KC760196	-	-
*P. cf. symmetrorchis*	*Clarias gariepinus*	Kenya	KF013171	KF013152	KF013162	-
*P. umblae*	*Pisidium hibernicum, Thymallus thymallus*	Norway	KP284110, KP284112, KP284109	-	KP284117, KJ740510	-
*P. vaili*	*Mulloidichthys flavolineatus*	Australia	KF013173	KF013155	KF013155	-
*P. wallacei*	-	Central Mexico	KT376714	-	-	-
*Phyllodistomum* sp.	*Macrobrachium dayanus, Perca fluviatilis, Epibulus insidiator, Cephalopholis boenak, Archocentrus centrarchus, Rhamdia nicaraguensis, Cyprinus carpio, Nodularia douglasiae, Profundulus labialis*	India, Russia, French Polynesia, Australia, Nicaragua, Japan, Mexico	OR269617*, KY307869, KF013179, KF013175	OR277452*	OR271917*, KY307886	OR420918, MW804299, MW804300, AB987943, AB987945, MW804303, MW804301

## Results

**Fig. 2:**
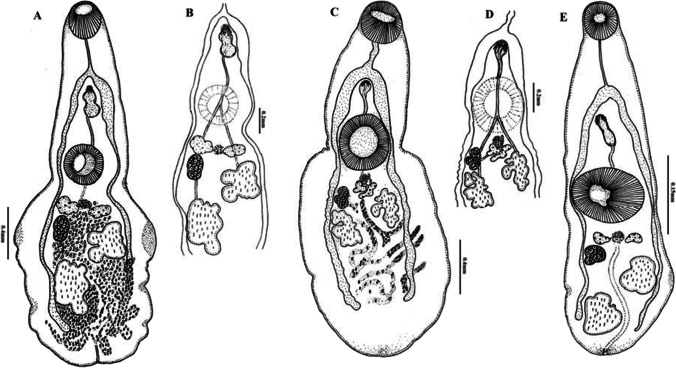
*Phyllodistomum* spp. found in the present study: **A** Adult *P. srivastava*, **B** detail of male and female reproductive complex of adult *P. srivastava,* **C** adult *P. parorchium*, **D** detail of male and female reproductive complex of adult *P. parorchium*, **E** metacercaria of *Phyllodistomum* sp. Note hind body with more or less crenated margin, elongated narrow esophagus, lobed testes positioned asymmetrically, and ovary with smooth margin in *P. srivastava* versus hind body with broad less crenulated margin, tubular esophagus, deeply lobed testes positioned almost symmetrically and lobulated ovary in *P. parorchium*

Based on their morphological features, the specimens of *Phyllodistomum* were identified as *P. srivastava*, *P. parorchium*, and one unknown *Phyllodistomum* metacercaria (Fig. 2). Details of the parasites found in the present study are provided in Table 3.

**Table 3:** Details of the hosts and parasites found in the present study

Host species	No. of hosts examined	Parasite, developmental stage (Voucher specimens)	Locality	Infected organ	No. of infected hosts	Parasites range	Total no. of parasites
Stinging catfish *Heteropneustes fossilis* (Bloch 1974)	25	*P. srivastava* Rai 1964, adult (W10415/1)	River Gomti, Lucknow, India	Urinary Bladder	14	2-3	32
Tank goby *Glossogobius giuris* (Ham, 1822)	18	*P. parorchium* Jaiswal 1957, adult (W10414/1)	River Gomti, Lucknow, India	Body cavity	9	1-2	18
Freshwater shrimp *Macrobrachium dayanus* Henderson, 1893	22	*Phyllodistomum* sp. metacercaria (W10413/1)	River Gomti, Lucknow, India	Hepatopancreas	10	8-10	86

### Morphological redescriptions

 Measurements of the specimens found in the present study (*n* = 10) are given in millimeters in Table 4.

**Table 4:** Comparative measurements (presented in mm) of *Phyllodistomum* spp. found in the present study with the closely related taxa

Taxa	*P. srivastava*	*P. srivastava*	*P. lucknowensis*	*P. parorchium*	*P. parorchium*	*Phyllodistomum* metacercaria	*P. srivastava* Rai 1964	*P. lucknowensis* Pandey 1970
Reference	Present study	Rai 1964	Pandey 1970	Present study	Jaiswal 1957	Present study	Rai 1964	Pandey 1970
Host	*Heteropneustes fossilis*	*Heteropneustes fossilis*	*Heteropneustes fossilis*	*Glossogobius giuris*	*Glossogobius giuris*	*Macrobrachium dayanus*	Shrimp	Shrimp
Body length	1.48-2.21	1.72-2.71	2.59-2.78	1.48-2.08	1.92-2.74	0.80–0.83	1.58–2.40	0.50-0.86
Body width	0.70-0.88	NS	1.28-1.34	0.80-0.84	1.10-1.13	0.22–0.25	1.05–1.76	0.23-0.41
Fore body length	0.72-0.76	0.86-1.36	NS	NA	NA	NA	NA	NA
Fore body width	0.52-0.58	0.45-0.70	NS	NA	NA	NA	NA	NA
Hind body length	0.76-1.20	0.87-1.41	NS	NA	NA	NA	NA	NA
Hind body width	0.82-1.10	0.78-1.24	NS	NA	NA	NA	NA	NA
Oral sucker	0.20-0.24 × 0.19-0.21	0.15-0.27 × 0.15-0.23	0.33-0.35 × 0.27-0.30	0.20-0.23 × 0.22-0.24	0.26-0.34 × 0.25	0.05–0.07 × 0.06–0.08	0.15–0.22 × 0.12–0.22	0.12-0.24
Esophagus length	0.15-0.20	0.12-0.20	0.15-0.28	0.15	0.09	0.11	0.10–0.23	0.25-0.35
Esophagus width	0.02-0.04	0.03-0.05	NS	NS	NS	NS	NS	NS
Ventral sucker	0.25-0.27 x 0.15-0.18	0.12-0.17 × 0.08-0.20	0.41-0.48 × NS	0.25-0.29 × .26-0.30	0.36-0.40 × 0.29	0.04–0.06 × 0.02–0.03	0.23–0.37 × 0.23–0.38	0.18-0.37
Anterior testis length	0.22-0.25 × 0.15-0.17	0.15-0.37 × 0.15-0.23	0.17-0.36 × 0.22-0.30	Left: 0.12-0.15 × 0.18-0.21	0.30-0.36 × 0.34-0.47	0.07–0.09 × 0.05–0.06	0.12–0.25 × 0.15–0.22	0.24-0.30x0.17-0.31
Posterior testis length	0.24-0.28 × 0.21-0.24	0.14-0.37 × 0.17-0.28	0.18-0.36 × 0.21-0.36	Right: 0.12- 0.15 × 0.21-0.24	0.28-0.29 × 0.30-0.36	0.09–0.10 × 0.05–0.07	0.16–0.25 × 0.12–0.27	0.20-0.31x0.15-0.34
Ovary	0.15-0.17 × 0.06-0.09	0.25-0.23	0.19-0.16	0.08-0.12 × 0.06-0.09	0.12 × 0.24	0.02–0.04 × 0.30-0.34	0.09–0.13 × 0.07–0.15	0.09-0.13x0.10-0.18
Vesicula seminalis length	0.19-0.21	0.05-0.08	NS	0.12-0.15	NS	0.04–0.06	0.043–0.05	NS
Vesicula seminalis width	0.07-0.09	0.05-0.10	NS	0.02-0.04	NS	0.02–0.03	0.04–0.07	NS
Left vitelline glands length	0.06-0.09	0.03-0.07	NS	0.02-0.04	NS	0.02–0.03	0.025–0.05	NS
Left vitelline glands width	0.11-0.13	0.13-0.25	NS	0.05-0.07	NS	0.01–0.02	0.10–0.21	NS
Right vitelline glands length	0.12-0.15	0.041-0.075	NS	0.02-0.04	NS	0.02–0.03	0.025–0.05	NS
Right vitelline glands width	0.04-0.07	0.15-0.217	NS	0.04-0.07	NS	0.01–0.02	0.125–0.217	NS
Eggs	0.01-0.03 × 0.01-0.03	0.032-0.040 × 0.014-0.024	0.052-0.072 × 0.031-0.035	0.01-0.03 × 0.01-0.03	0.27-0.35 × 0.14-0.25	NA	NA	NA

***P. srivastava*** **Rai,** 1964 (Fig. 1a)

**Description**: Body aspinose, elongated, pyriform, hind body with more or less crenated margin. Oral sucker globular, subterminal or terminal, pharynx absent, esophagus straight, thin, long, bifurcates into two intestinal ceca, extending up to hind end of the body. Ventral sucker larger then oral sucker situated in the middle of the body. Testes deeply lobed, oblique, intercecal, post-equatorial, tandem, vas deferens leading to bipartite vesicula seminalis, ejaculatory duct short, inconspicuous, opening into the genital atrium, cirrus lacking. Ovary subglobular or lobed, intercecal, anterior to left testis. Vitellaria paired compact masses, slightly lobed, round or oval, between ventral sucker and ovary. Laurer’s canal not observed. Mehlis’ gland small, between vitelline masses. Uterus extensively coiled, occupying entire hind body, intercecal or extracecal, occupying posterior half of hind body, uterine coils extending posteriorly beyond ceca, filled with eggs, genital pore sub-median. Excretory bladder tubular, opening by a median excretory aperture in the caudal notch. Eggs small, operculated. 

***P. parorchium*** **Jaiswal,** 1957 (Fig. 1b)

**Description**: Body aspinose, less crenulated margin, broad, oral sucker globular, subterminal or terminal, ventral sucker rounded, equatorial, larger than an oral sucker, pharynx absent, esophagus tubular, leading into two broad intestinal ceca, extending up to the hind region of the body. Testes two, deeply lobed, oblique, intercecal, anterior testis situated close to vitellarium, posterior testis close to ovary, vesicula seminalis free in the parenchyma, opening by a short duct at genital pore. Cirrus sac absent, seminal vesicle single chambered, just posterior to intestinal bifurcation, before opening into genital atrium, anterior end surrounded by prostatic cells. Ejaculatory duct short. Genital pore median at the level of intestinal bifurcation, sperms visible in the uterus. Ovary trilobed, anterior and oblique to posterior testis. Vitelline massed two, deeply lobed with irregular margins, behind ventral sucker. Mehlis’ gland small, between vitelline masses. Laurer’s canal well developed. Uterus highly coiled, intercecal or extracecal, descending limb of uterus occupying posterior half of hind body, ascending limb of uterus running forward dorsal to ventral sucker and narrows to form metraterm which opens into genital pore. Excretory bladder tubular, run in a zig-zag manner, up to ventral sucker, expanding into a sac-like structure and excretory pore situated at the posterior end of the body. Eggs oval, thin, light brown and operculated.

**Metacercaria of** *P. srivastava* **Rai,** 1964 (Fig. 1c)

**Description**: Body elongated, aspinose, pyriform, oral sucker globular, subterminal or terminal, ventral sucker equatorial, larger than an oral sucker, pharynx absent, esophagus straight, long, bifurcates into two broad intestinal caeca, extending up to the hind end of the body. Testes two, lobed, intercecal, oblique, almost similar size, in posterior third of hind body, vas deferens leading to bipartite seminal vesicle, ejaculatory duct short, inconspicuous, opening into the genital atrium, cirrus lacking. Genital pore situated slightly away from intestinal bifurcation. Ovary subglobular or lobed, behind left or right vitellarium. Vitelline masses paired, compact, slightly lobed, posterior to ventral sucker, Mehlis’ gland dorsal, posterior to ventral sucker, between vitelline masses. Laurer’s canal not observed. Excretory vesicle long, tubular, lies between testes, opening by the median excretory aperture.

### Molecular results

Sequences of the 28S (374-384 bp long), ITS1 (973-982 bp long), ITS2 (433-454 bp long), and CoxI (388-433 bp long) were obtained successfully for all taxa in the present study. The nucleotide sequences obtained in this study were submitted to NCBI/GenBank database (Table 2).

Alignment and comparison of the sequences between the metacercaria in the present study and adult *P. srivastava* revealed 0.6%, 0.0%, 0.0%, and 0.2% bp difference in 28S, ITS1, ITS2, and Cox1 regions, respectively. The bp difference for these regions, between metacercaria in the present study and adult *P. parorchium,* were 2.7%, 3.8%, 3.7%, and 3.7%, respectively, which was within the range observed for the bp difference between distinct species, *P. srivastava* and *P. parorchium,* 2.9%, 4.0%, 3.8%, and 6.3, respectively.

**Fig. 3:**
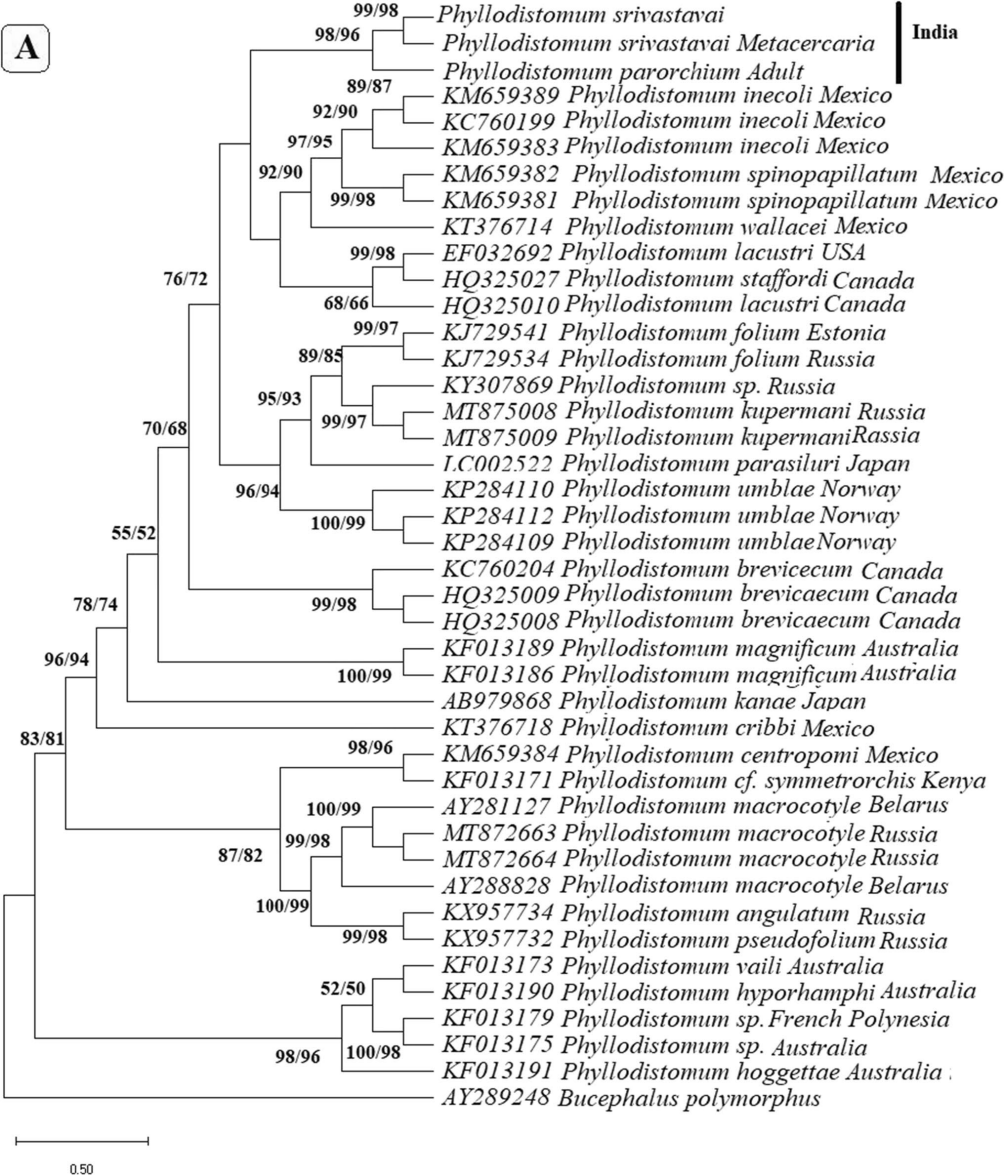

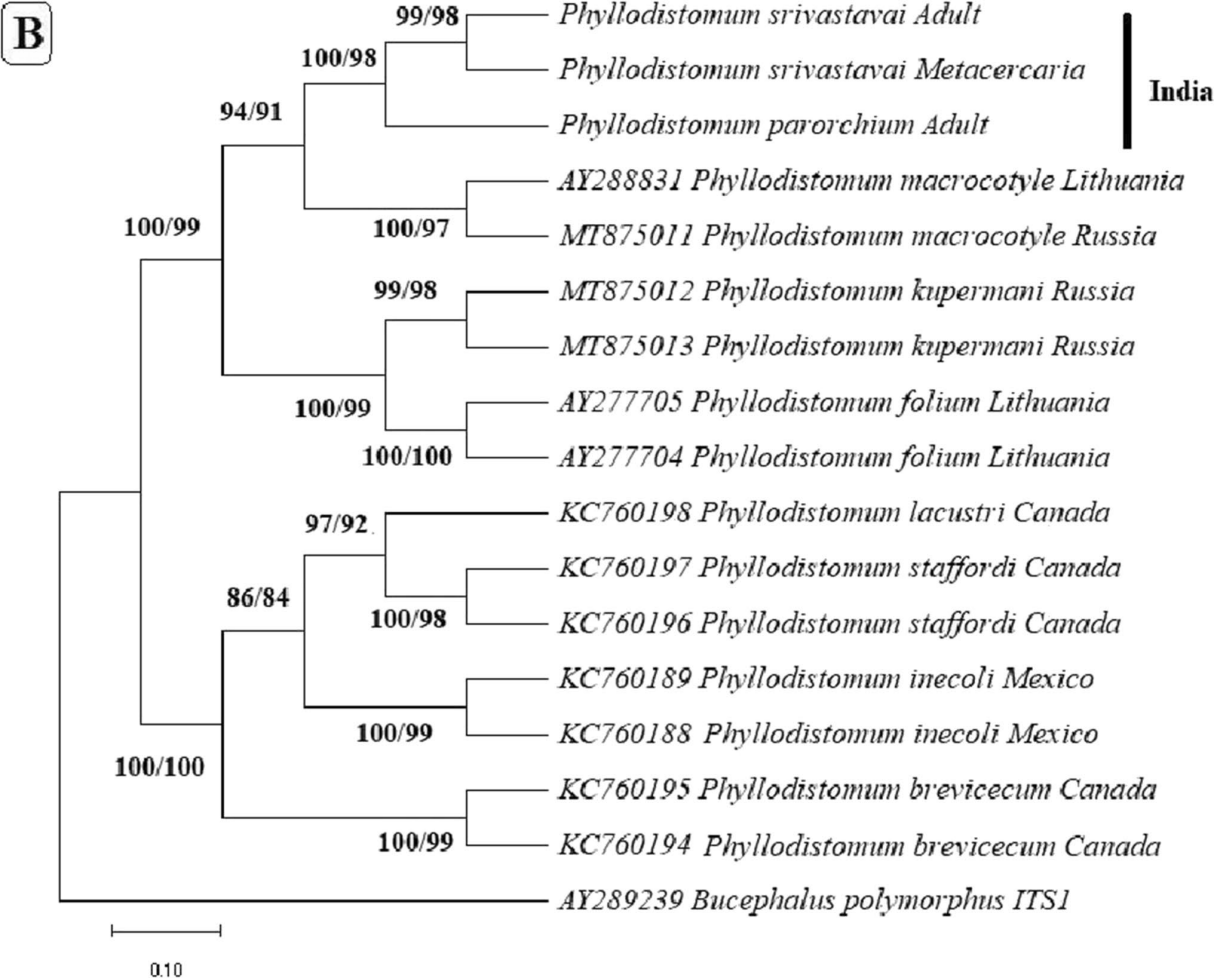

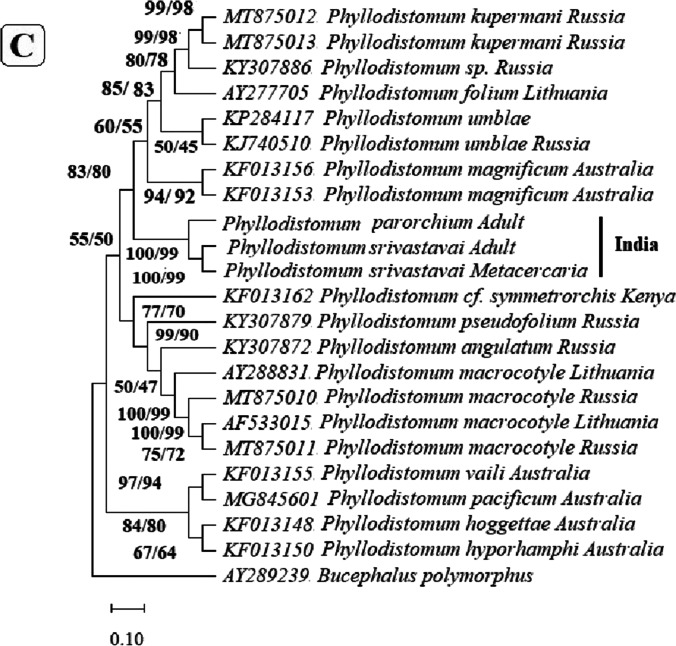

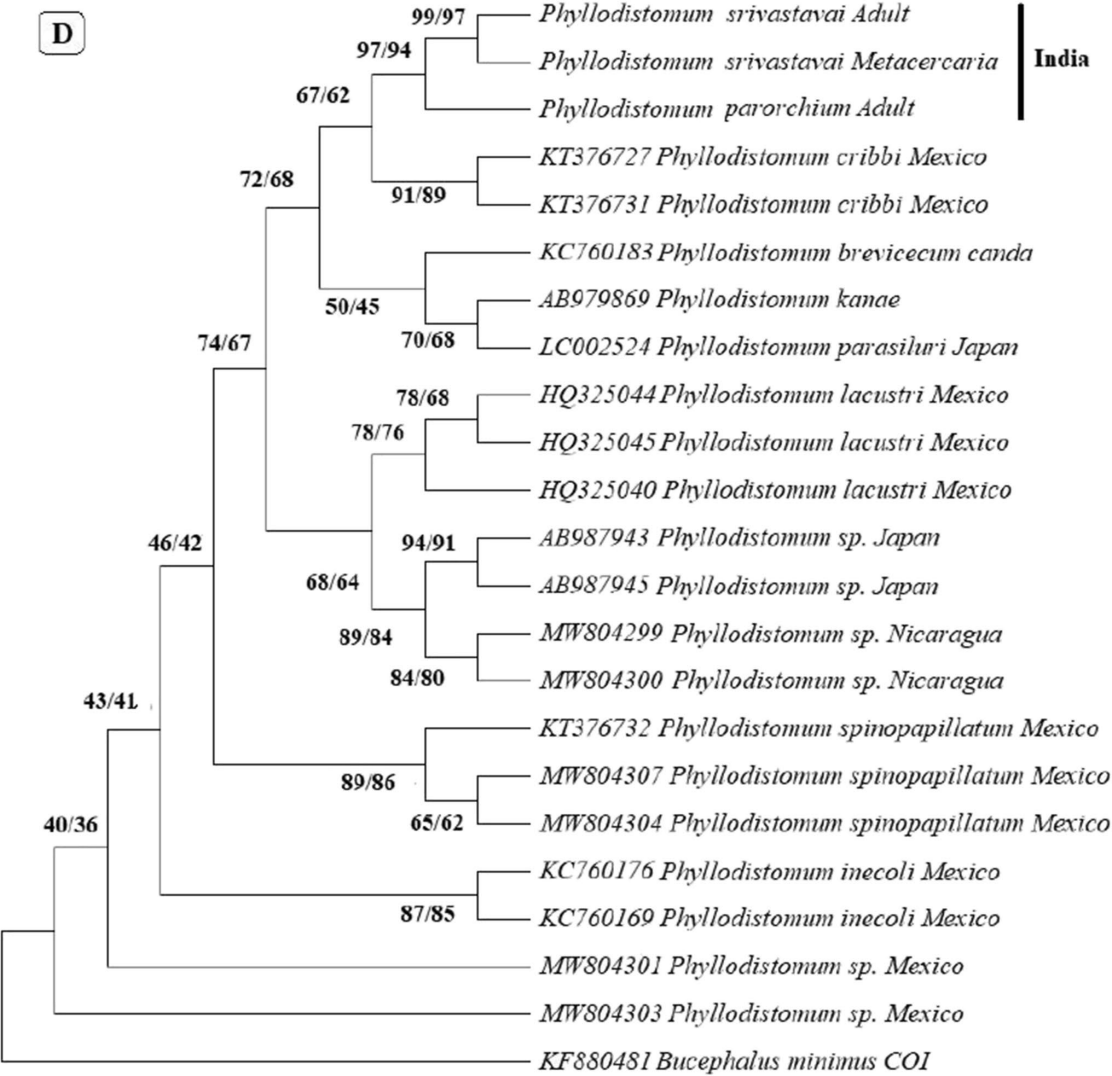
Neighbor joining and maximum likelihood phylogenetic trees built based on the sequences of **A** 28S, **B** ITS-1, **C** ITS2, and **D** Mt CoxI gene of *Phyllodistomum* spp. (*P. srivastava*, *P. parorchium*) and one *Phyllodistomum* metacercaria using the member of the genus *Phyllodistomum*. The number preceding the GenBank accession numbers for their 28S r RNA gene sequences. The numbers of the internodes are NJ bootstrap values (above) and ML bootstrap values (below)

In the phylogenetic trees built based on these regions (Fig. 3), metacercaria found in the present study consistently grouped with *P. srivastava,* with *P. parorchium* grouping closely but distinctly from them.

## Discussion

The taxonomic status of two species of *Phyllodistomum* in India was investigated through a comprehensive analysis combining morphology and molecular techniques. The morphological characteristics of the specimens closely resembled the original description of *P. srivastava*, in terms of the ratio of suckers, diagonal arrangement of testes, bipartite vesicula seminalis, and paired vitellaria (Table 4). However, some minor differences in body shape were observed, which could potentially be attributed to variations in fixation and mounting techniques used during the study. A consistent finding in our measurements was that the specimens appeared smaller compared to those documented by Rai (1964). Additionally, Rai (1964) reported presence of papillae in live specimen which gradually disappeared after fixation. This observation underscores the importance of considering the impact of fixation on morphological features, as it could lead to changes or loss of certain characteristics, potentially affecting the accuracy of taxonomic comparisons.

Cribb (1987) described *Pseudophyllodistomum johnstoni* which included five previously described species of *Phyllodistomum*. These species were reclassified under the new genus as follows: *P. macrobrachicola* (Yamaguti 1934) comb. nov., *P. lesteri* (Wu, 1938) comb. nov., *P. srivastava* (Rai 1964) comb. nov., *P. lucknowense* (Pandey 1970) comb. nov., and *P. mingense* (Tang, 1985) comb. nov. However, there are clear distinguishing features between *P. johnstoni* and *P. srivastava*. *P. johnstoni* which can be readily distinguished from *P. srivastava* by shape and size of body, its nearly equal oral and ventral sucker, single chambered seminal vesicle, and saccular excretory bladder. Additionally, *P. johnstoni* occur as a parasite of the urinary bladder of the Australian freshwater fish *Leiopotherapon unicolor*, whereas the *P. srivastava* recovered from the Indian cat fish *Heteropneustes fossilis* (Bloch 1974).

In case of *P. parorchium,* the present specimens closely resembled the description given by Jaiswal (1957) in terms of the ratio of suckers, position of genital pore, shape of testes, ovary, and vitellaria (Table 4). However, there are significant distinguishing traits between *P. srivastava* and *P. parorchium*. These characteristics include shape and size of body, absence of a crenulated margin on the hind body, presence of a large ventral sucker, a single chambered seminal vesicle, and deeply lobed vitellarium. Pandey (1970) described metacercaria od *P. lucknowense*, which was found in the same host, *Macrobrachium dayanum* (Henderson). The metacercaria *P. lucknowense* differs from *P. srivastava* in several aspects, such as the presence of a crenulated body margin, a spinose body, a saccular vesicula seminalis, and shape of the testes. Additionally, the present specimens of *P. srivastava* also differ from *P. lucknowense* Pandey 1970 by spinose body, saccular vesicula seminalis, and size of eggs. So far, only two metacercariae of *Phyllodistomum*, namely, *P. srivastava* and *P. lucknowense*, have been reported in the Indian subcontinent from shrimp, and their adults were obtained experimentally by feeding on *Heteropneustes fossilis* (Rai 1964; Pandey 1970)*.* The metacercaria and adult of *P. srivastava* were found in the same locality and recovered from naturally infected hosts. The development of adult *P. srivastava* in fish is attributed to carnivorous nature of the fish host, *H. fossilis*, and the availability of insects and crustacean preys in its vicinity.

The comparative morphology of the present metacercaria showed a close resemblance to the adult of *P. srivastava* Rai 1964, including the ratio of suckers, the arrangement of testes, bipartite vesicula seminalis, and paired vitelline masses, but they differ from *P. parorchium* which has symmetrical testes, less crenulated margin, and deeply lobed vitelline follicles.

The morphological studies were supported by molecular findings as well. *Phyllodistomum* over the last decade, particularly for 28S rRNA, ITS 1, and CoxI sequences. Over the last decade, the increasing availability of genetic resources has greatly enhanced our understanding of the diversity and phylogenetic relationships of gorgoderid trematodes (Razo-Mendivil et al. 2013; de León et al. 2015a; de León et al. 2015b; Pinacho-Pinacho et al. 2021).

Regarding the comparison of base pair (bp) differences for different regions, our results align with previous studies. Razo-Mendivil et al. (2013) reported no intra-specific variation in isolates of *P. inecoli* and *P. brevicecum* for 28S and ITS1, whereas inter-specific variation ranged from 3.2 to 4.4% for 28S r DNA and from 8.3 to 14.7% for ITS1. In contrast, Petkevičiūtė et al. (2015) observed 0.3% intra-specific variation for *P. folium* and 0.5% for *P. macrocotyle* in 28S sequences. European species of *Phyllodistomum,* on the other hand, exhibited high inter-specific variation (8.5% to 14%). In another study by Petkevičiūtė et al. (2015), no intra-specific variation was found in 28S sequences of different isolates of European species *P. folium.*

Similarly, de León et al. (2015b) identified genetic variation in the 28S rRNA gene between *P. spinopapillatum* and *P. inecoli*, ranging from 0.8 to 1.0% and between *P. lacustri* and *P. staffordi* ranging from 3.8 to 4.0%. Petkevičiūtė et al. (2020) identified genetic variation in the 28S rRNA gene between *P. kupermani* and *P. folium,* ranging from 1.1 to 1.3%, whereas 1.2 to 1.4% for ITS2; while it was 1.8% for *P. umblae* for 28S rRNA and 1.2% for ITS2. Pinacho-Pinacho et al. (2021) identified interspecific of CoxI sequence between *P. simoni* and *P. inecoli* ranging from 5.1 to 6.4%, while 6.5–10.7% for *P. spinopapillatum*.

In general, the intra-specific variation within the ITS region and mt CoxI is generally low, but consistent inter-specific differences exist (Nolan and Cribb 2005; Pinacho-Pinacho et al. 2021). To obtain a clear understanding of exact number of *Phyllodistomum* species in India, further studies are necessary. These studies should encompass both morphological and molecular analyses of metacercariae and adults, which will significantly contribute to the phylogenetic revision of gorgoderids. The combination of these approaches will provide valuable insights into the taxonomy and evolutionary relationships of these trematode species. Certain parasites with indirect life cycles possess the capability to manipulate the behaviour of their hosts (Freire et al., 2022), thereby aiding in their transmission to definitive hosts. Given the discovery of *Phyllodistomum *metacercariae in shrimp and adults in fish during the current study, an interesting area for future research would involve exploring the potential impact of *Phyllodistomum *infection on shrimp behaviour. Certain digenean parasites, including *Clinostomum* and *Paramphistomum*, are recognized for their migration through gastrointestinal tissues before reaching adulthood in the host's stomach (Rolfe et al. 1994, Shamsi et al. 2013). This process leads to pathogenic effects in definitive hosts. Hence, an additional prospective area for future research lies in examining the health consequences and pathogenicity induced by these parasites in their fish hosts.

## Supplementary information

ESM 1 (JPG 1344 kb)

ESM 2 (JPG 1536 kb)

ESM 3 (JPG 2246 kb)

ESM 4 (JPG 1215 kb)

ESM 5 (JPG 1653 kb)

ESM 6 (JPG 1319 kb)

## Data Availability

Sequences are publicly available in GenBank.
