# Supplementary figures and images for: Genetic characterization and phylogenetic relationships of Phyllodistomum parasites in Indian subcontinent: insights from freshwater fish and shrimp hosts
Source: Parasitol Res. 2023 Aug 23;122(10):2301–15. doi: 10.1007/s00436-023-07930-3 (PMC10495520; doi:10.1007/s00436-023-07930-3)

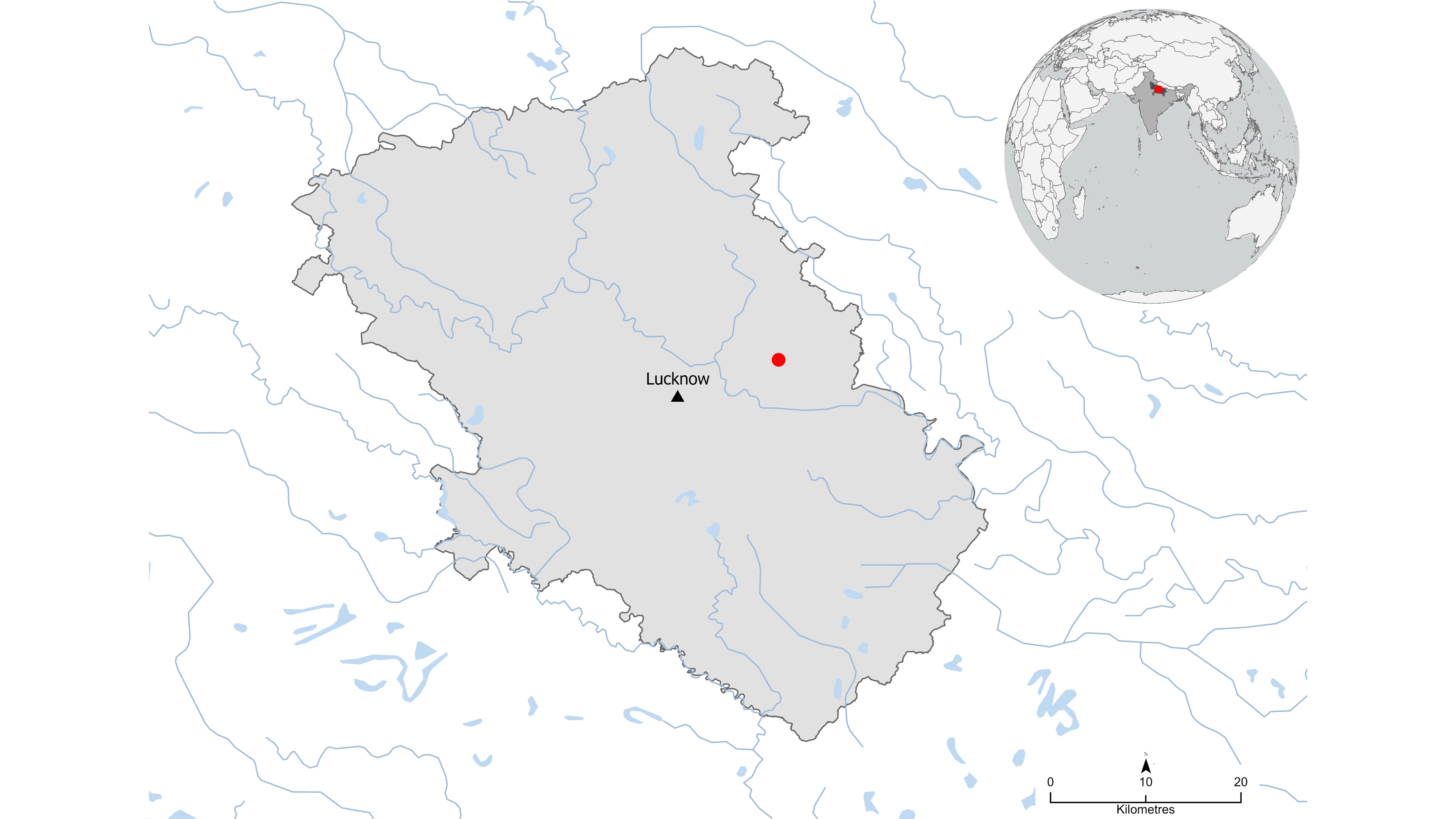

Supplement: Supplementary file 1 — (JPG 1344 kb) [file 436_2023_7930_MOESM1_ESM.jpg]

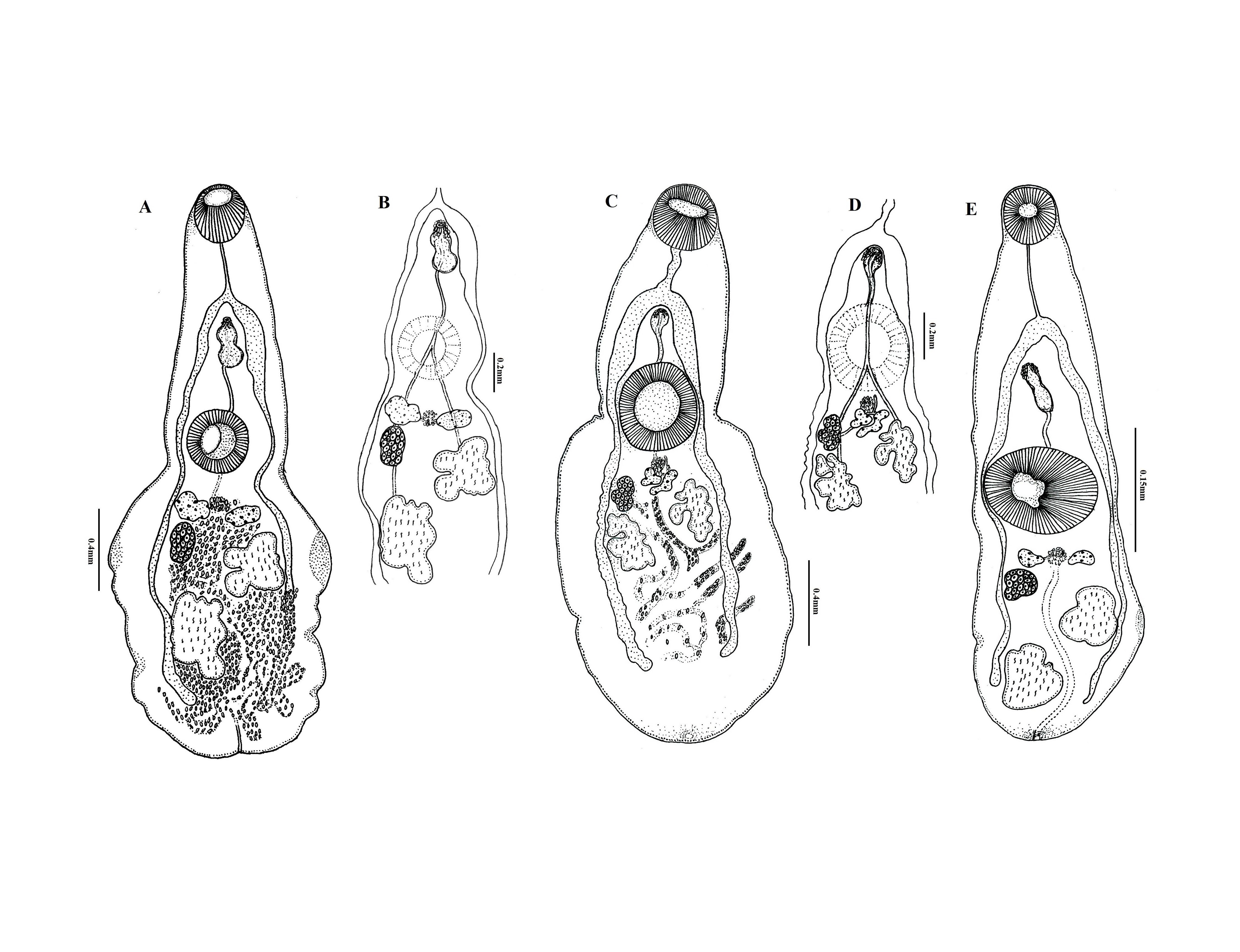

Supplement: Supplementary file 2 — (JPG 1536 kb) [file 436_2023_7930_MOESM2_ESM.jpg]

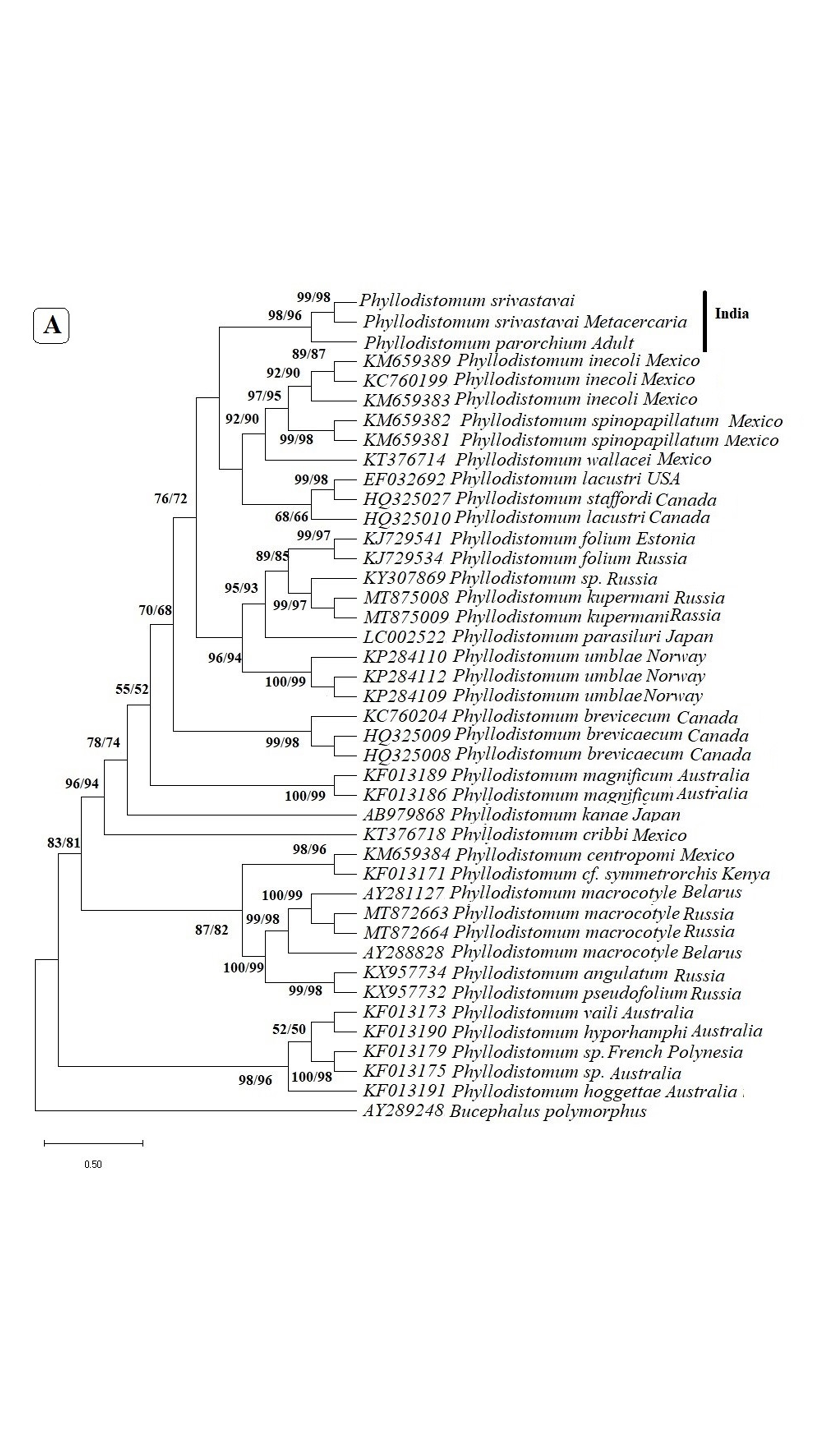

Supplement: Supplementary file 3 — (JPG 2246 kb) [file 436_2023_7930_MOESM3_ESM.jpg]

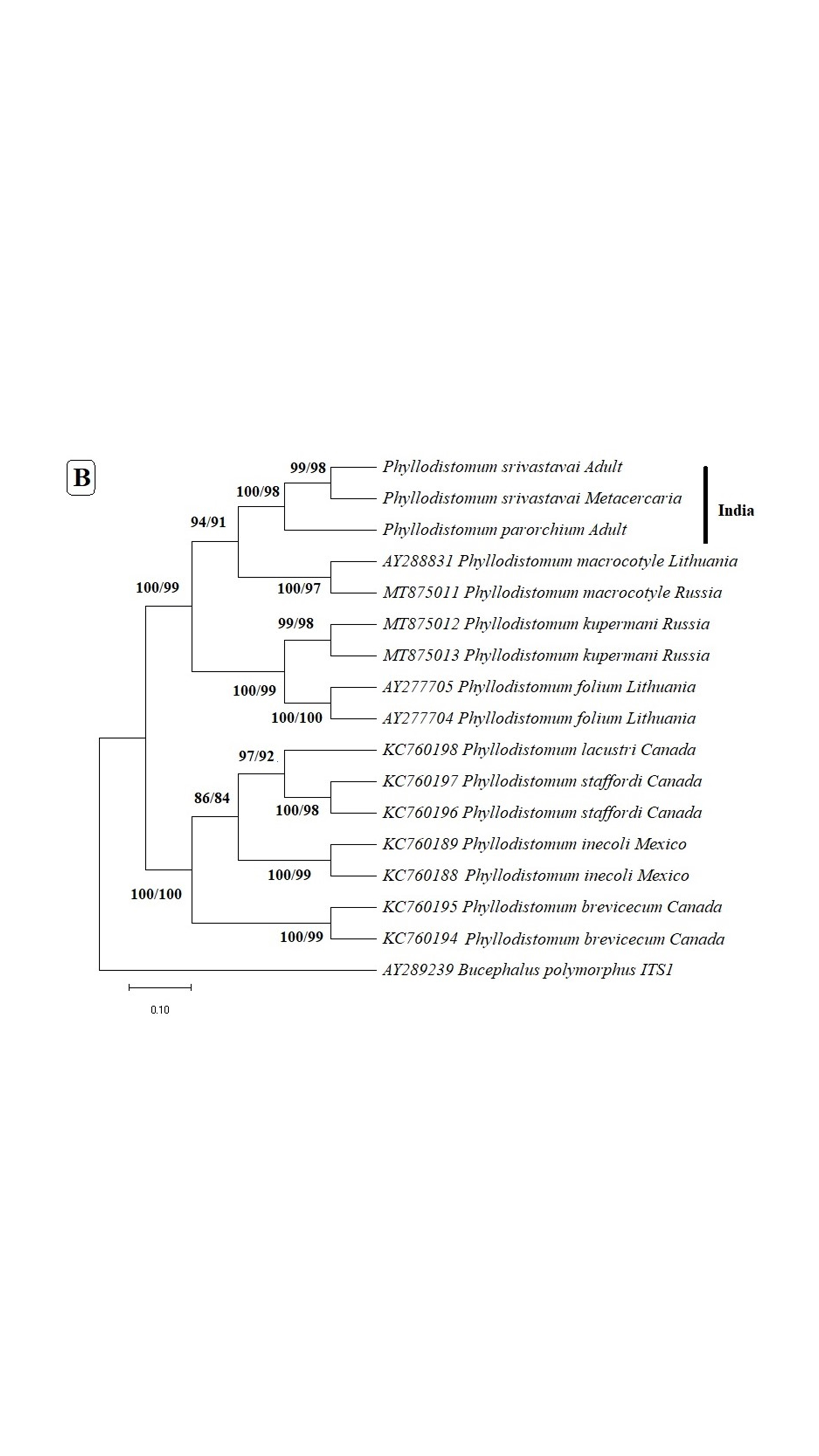

Supplement: Supplementary file 4 — (JPG 1215 kb) [file 436_2023_7930_MOESM4_ESM.jpg]

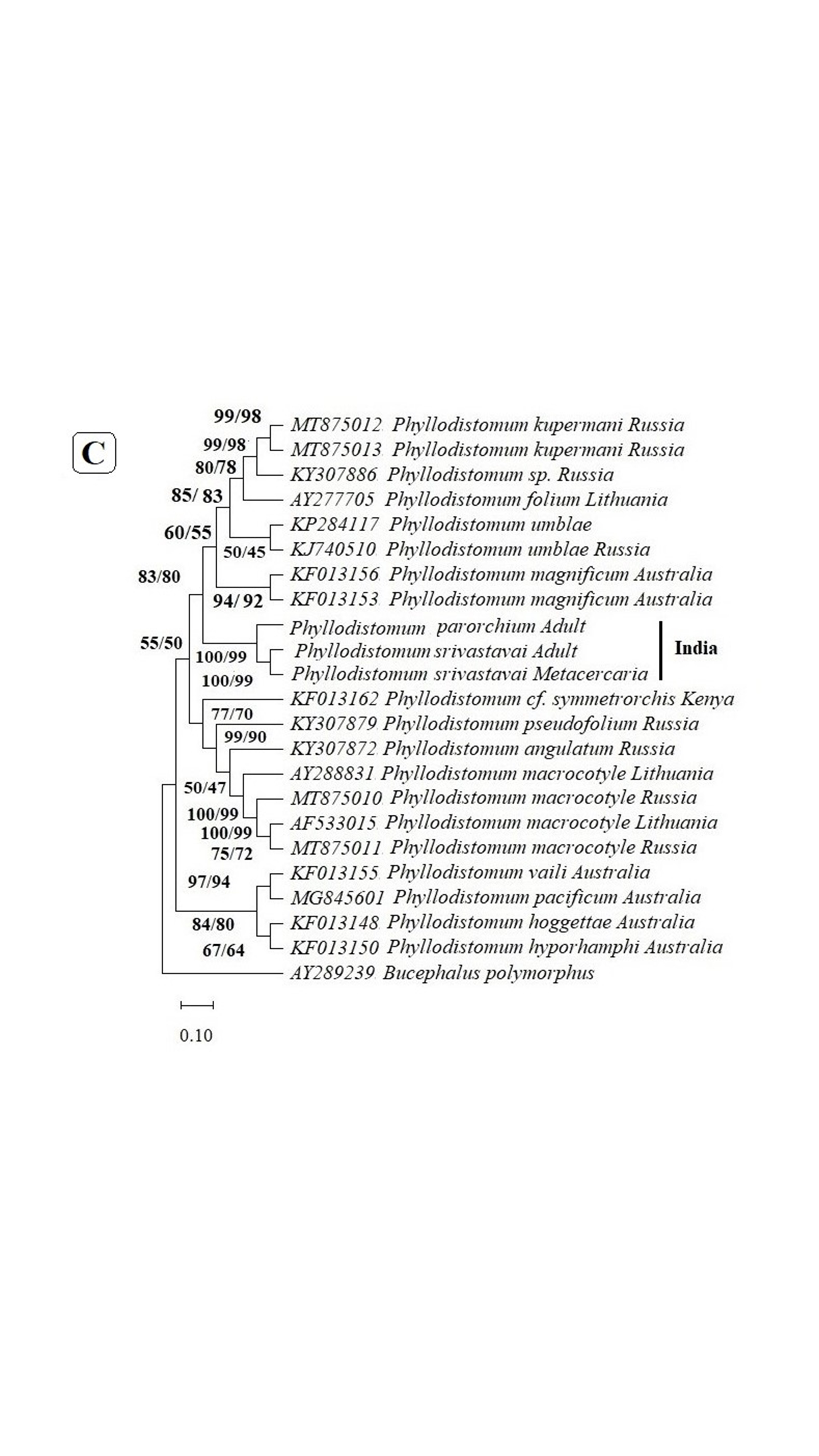

Supplement: Supplementary file 5 — (JPG 1653 kb) [file 436_2023_7930_MOESM5_ESM.jpg]

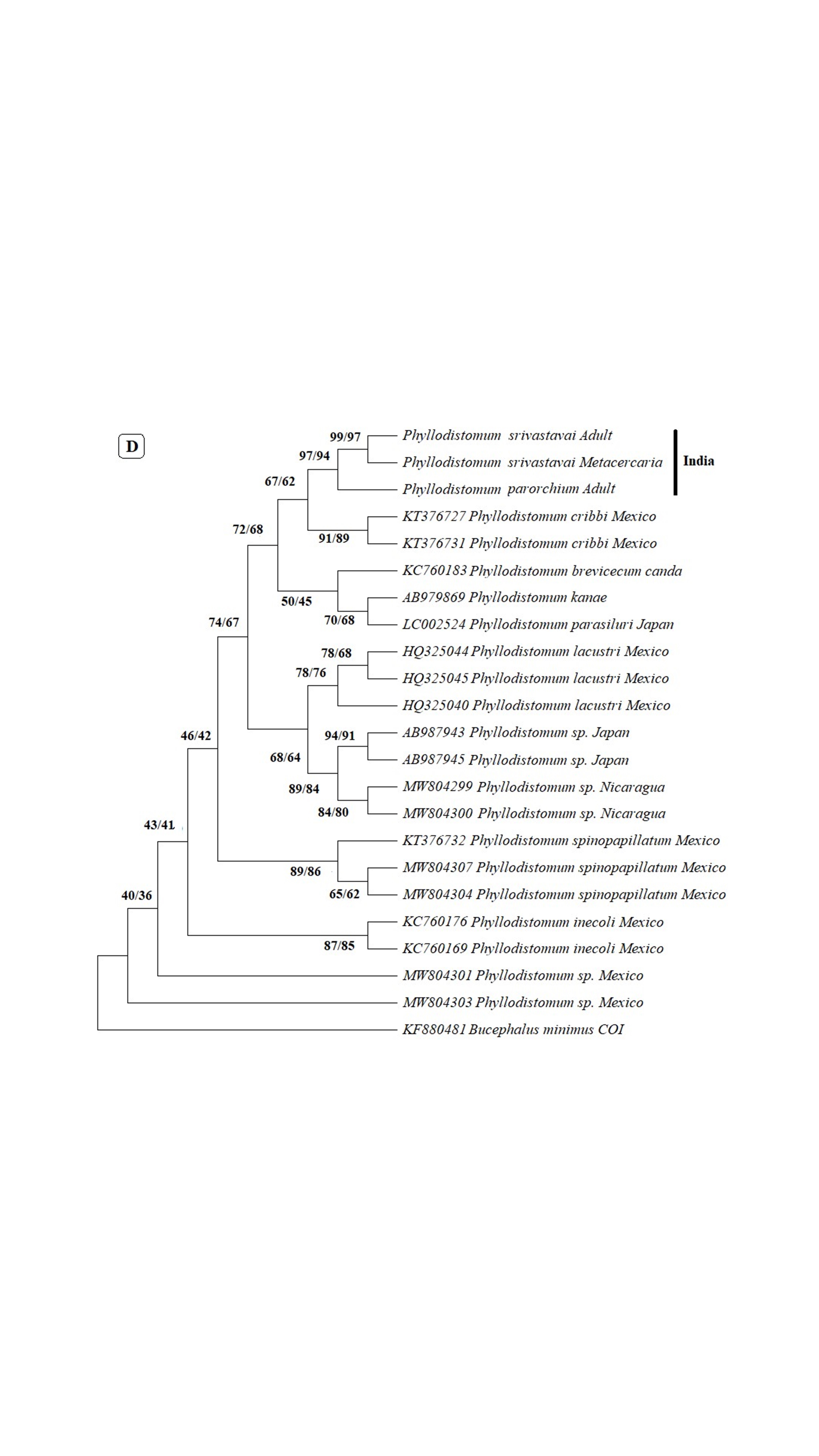

Supplement: Supplementary file 6 — (JPG 1319 kb) [file 436_2023_7930_MOESM6_ESM.jpg]
